# The Illinois State Board of Health Report

**Published:** 1889-03

**Authors:** 


### EDITORIAL.

### THE ILLINOIS STATE BOARD OF HEALTH REPORT.

The fifth "Report on Medical Education, Medical Colleges and the Regulation of the Practice of Medicine in the United States and Canada: 1765-1889," has been recently issued by the Secretary of the Illinois State Board of Health. There are many good things to be said of this Report. In the first place, it should be a matter of self-congratulation on the part of the people of the State that this is not only the only report of the same or similar nature issued in this country, but it is recognized as a valuable work of reference in all parts of the world. Chiefly by its work in raising the standard of medical education, in exposing "diploma mills," and in tracing up and prosecuting quackery, the Illinois State Board of Health has made itself a power that is felt over the United States and Canada, and even in far-away Australia.

It is pleasing to be able to say also, of this Report, that it is the most encouraging one ever issued by the Board. It shows that there is more promise of the raising of medical education to the desired standard than ever before, and that the Board and they that have spent so much time and written so much in the cause of higher medical education have not labored in vain. The Report says: "As to medical education in this country, the general drift of opinion to-day is decidedly in favor of the extension of the period of medical study to four years, and of attendance upon lectures to three full terms, with ample hospital practice and clinical instruction."

It is well known that the Board has passed the following resolution in regard to four years of study and attendance upon three courses of lectures:

"That the phrase, 'medical colleges in good standing,' in the first section of the 'Act to regulate the Practice of Medicine' in the State of Illinois, approved June 16, 1887, is hereby defined to include only those colleges, which shall, after the sessions of 1890 91 require four years of professional study including any time spent with a preceptor, and three regular courses of lectures, as conditions of graduation, and shall otherwise conform to the Schedule of Minimum Requirements heretofore adopted by the Board." To the "four years'" clause we have the serious objection that the time spent in so-called professional study before the student enters college is practically wasted time. As a matter of fact, preceptors do not teach students; the student may register under a preceptor, and obtain a certificate of one year's preceptorship, when he has spent his time entirely otherwise than in professional study. Half a century ago the preceptor did teach his student; now, in the vast majority of cases, he does not. The rule of the Board, therefore, is an incentive to deception. It would have been better had the Board left out the four years' clause, or else demanded four years' attendance upon lectures and at clinical work. The time for demanding four years of study in the colleges has not come, we think; meanwhile it is best, in our opinion, to demand simply three years of study in the colleges, without reference to so-called "previous study under a preceptor."

While we may not be in accord with "the general drift of opinion to-day," we believe that we stand on firm ground in this matter. Four years of college work is inevitable, and the sooner we get to it the better for the medical profession and for the people. Clinical teaching, which is by far too much neglected in this country, is neglected for want of time. "The difficulty," says Professor Wm. Osler (*Journal of the American Medical Association*, March 23, 1889), "lies entirely in the short three session course. The students, teachers and patients are here, and there is no reason why Philadelphia, New York, Boston, Baltimore, Chicago, St. Louis and Cincinnati should not have clinical teaching just as thorough, and just as systematic as is given to-day in Edinburgh, or on this continent, in Montreal. Large classes, in large cities with ample hospital facilities, can be perfectly well managed. The lack of a fourth year is the only obstacle."

The rule of the Board to which we object is a step in the direction of a four years' course. Already a number of colleges have announced that they will conform to this rule. We have no objection to proper steps in the direction of a four years' course, but our objection is to the manner of taking the step.

Unfortunately there are some colleges that are not affected by the regulations of the Illinois Board. We refer more particularly to some in the South and in the far West, from which graduates do not come before the Illinois State Board of Health. Unfortunately, too, these colleges do not fall under the restricting influences of any other similar board.

Probably one of the strongest arguments in favor of a higher standard of medical education to be found in the Report is the fact that of the 267 medical institutions (not including preparatory schools) embraced in the Report, only 131 are now in existence. And the fact that Canada has one extinct medical school, and the United States 130, seems sufficient to show that the methods of medical education are better in Canada than in the United States; and it would be a matter of congratulation if more were extinct, since the *raison d'être* of many of them is not apparent. In Germany twenty medical schools are sufficient for the forty-five million people of the country, and for students from all over the world; but in this country ninety-three, it would appear, are required for sixty million people. One thing, however, seems certain: there is an increasing demand for better work on the part of the colleges; and the colleges that do not meet this demand must close their doors. The Report shows that there is an increased number of medical schools that have recognized their duties, but there are some that seem to be still "wedded to as low a standard as is at all compatible with even scant recognition by the medical profession."

On the whole, as has been said already, this is a very encouraging Report.

